# Can Attention Be Confined to Just Part of a Moving Object? Revisiting Target-Distractor Merging in Multiple Object Tracking

**DOI:** 10.1371/journal.pone.0041491

**Published:** 2012-07-30

**Authors:** Piers D. Howe, Natalie C. Incledon, Daniel R. Little

**Affiliations:** School of Psychological Sciences, University of Melbourne, Melbourne, Australia; Goldsmiths, University of London, United Kingdom

## Abstract

While it was initially thought that attention was space-based, more recent work has shown that attention can also be object-based, in that observers find it easier to attend to different parts of the same object than to different parts of different objects. Such studies have shown that attention more easily spreads throughout an object than between objects. However, it is not known to what extent attention can be confined to just part of an object and to what extent attending to part of an object necessarily causes the entire object to be attended. We have investigated this question in the context of the multiple object tracking paradigm in which subjects are shown a scene containing a number of identical moving objects and asked to mentally track a subset of them, the targets, while not tracking the remainder, the distractors. Previous work has shown that joining each target to a distractor by a solid connector so that each target-distractor pair forms a single physical object, a technique known as target-distractor merging, makes it hard to track the targets, suggesting that attention cannot be restricted to just parts of objects. However, in that study the target-distractor pairs continuously changed length, which in itself would have made tracking difficult. Here we show that it remains difficult to track the targets even when the target-distractor pairs do not change length and even when the targets can be differentiated from the connectors that join them to the distractors. Our experiments suggest that it is hard to confine attention to just parts of objects, at least in the case of moving objects.

## Introduction

Attention plays a vital role in visual cognition. It allows us to select some stimuli for preferential processing, while ignoring the rest, thereby preventing us from becoming overwhelmed [1], [2]. Initially, it was assumed that attention operated by selecting particular locations in space to receive enhanced processing [3]–[5]. More recently, it has been shown that attention can also operate in an object-based manner, more readily spreading throughout an object than crossing object boundaries [6]–[9]. For example, one study had observers report whether two parts of an object were identical [7]. Observers were quicker to respond if both parts belonged to the same object as opposed to when they belonged to different objects, despite the separation between the object parts being the same in both conditions.

While the above studies have shown that it is easier to attend to multiple parts of the same object, they did not investigate the extent to which attention automatically spreads to other parts of an object when the observer attempts to attend to just one part of an object. Scholl et al. [10] investigated this issue using a multiple object tracking (MOT) paradigm [11].

**Figure 1:**
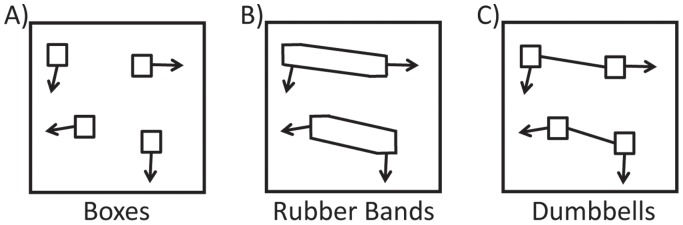
Some of the stimuli used by Scholl et al (2001). A) The boxes condition (i.e. the baseline condition). B) The rubber bands condition. C) The dumbbells condition.

In a baseline condition, referred to as the “boxes” condition, there were eight identical hollow squares, four of which were briefly highlighted to indicate that these were the targets to be tracked (Figure 1a). Then the highlighting disappeared, all the squares became identical and moved randomly about the display before coming to a halt. The observer then used a computer mouse to identify the four targets. Because the squares were all identical during the movement phase, the observer could identify the targets only if he had mentally tracked them. Accuracy was defined as the average number of targets correctly identified. For this condition accuracy was very high with observers often being able to track all four targets correctly.

A second condition, the “rubber bands” condition, was identical to the baseline condition except that each target was joined to a distractor by two parallel lines, so that each target-distractor pair appeared to form a single object with the target and distractor at opposite ends of this oblong (Figure 1b). We shall refer to the act of joining a target to a distractor via a connector as target-distractor merging. As before, the targets and distractors moved independently of each other. Accuracy was substantially reduced in this condition in comparison to the baseline condition. This was taken as evidence that in the rubber bands condition observers had difficulty confining their attention to the targets because attention tended to spread throughout the entire target-distractor pair, thereby reducing the tracking accuracy [10].

While Scholl et al.’s findings are impressive, there is an alternative explanation. Since the targets and distractors in the rubber bands condition were moving independently, the resultant target-distractor oblongs continuously elongated and contracted. A subsequent study has shown that observers find it difficult to track objects that elongate and contract, even when they do not have the additional task of having to keep track of just one end of each object [12].

VanMarle and Scholl [12] had observers perform a multiple object tracking task. There were two conditions of particular interest: The “objects” condition and the “slinky” condition. Their objects condition was very similar to the baseline condition of Scholl et al. [10]. The observer saw eight squares and was required to keep track of four of them. Performance was very high (89%). Conversely, in the slinky condition the objects were rectangles that moved in a non-rigid manner. Each rectangle would first anchor one end and then extend itself so that its other end would move to another point on the screen. Then the front of the rectangle would become fixed and the rectangle would then contract thereby bringing its rear end to the front end, at which point the motion sequence would repeat. In this way, the rectangles moved about the monitor in a manner that resembled slinky springs. As before, there were eight objects and the observers were required to keep track of four of them. Observers found this task to be very difficult and tracking performance was poor (69%), only slightly above chance performance (62.5%). By performing a series of control experiments it was concluded that the difficulty in tracking the rectangles in the slinky condition was due to them continuously changing their lengths. Thus, the tracking difficulty experienced by the observers in the rubber bands condition of Scholl et al. may have also been due to those objects continuously changing their length and not due to the difficulty of confining attention to the correct end of each oblong as was assumed by Scholl et al.

In our first experiment we addressed this potential confound by replicating the baseline and rubber bands conditions of Scholl et al. [10] but with the constraint that each target-distractor pair had a fixed length. We discovered that although this manipulation made it somewhat easier to track the targets in the rubber bands condition (i.e. the target-distractor merged condition), tracking accuracy was still below that in the baseline condition where the target and distractors were not connected. This showed that target-distractor merging per se can reduce tracking ability. This supports the Scholl et al. assertion that in the merged condition observers have difficulty restricting their attention to just the targets.

In Experiment 2, we investigated under what circumstances target-distractor merging can reduce tracking performance. Scholl et al. [10] ran several target-distractor merged conditions, yet in some of them observers found it easy to track the targets. For example, in one condition each target was joined to a distractor via a single line, so the resultant target-distractor pairs resembled “dumbbells” (Figure 1c). Scholl et al. found that tracking accuracy in this condition was only slightly less than that in the baseline condition in which the targets were not joined to the distractors (Figure 1a). They suggested that the reason why tracking accuracy was much greater in the dumbbells condition (Figure 1c) than in the rubber bands condition (Figure 1b) was that in the dumbbells condition the targets could be differentiated from the connecting bars, which helped to confine attention to just the targets.

Experiment 2 was designed to test this hypothesis. In this experiment we kept the targets and distractors black but made the connecting bars white, thereby making it possible to differentiate the targets from the connecting bars. According to the Scholl et al. hypothesis [10], it should now have been easier to confine attention to the targets, so tracking accuracy in this condition should have been similar to that in the baseline condition, whereby the target and distractors were not physically connected. We found this not to be the case, with tracking accuracy being still much higher in the baseline condition.

The result of Experiment 3 surprised us, so we decided to replicate the Scholl et al. [10] dumbbells condition to verify that we could obtain the same result as them, i.e. that tracking accuracy in the dumbbells condition was approximately equal to that in the baseline condition. In fact, we found that tracking accuracy in the dumbbells condition was significantly worse than that in the baseline condition where the targets and the distractors were not physically connected.

Our first three experiments demonstrated that target-distractor merging effects are robust. Tracking accuracy decreased whenever the targets were joined to the distractors via a connector, even when the targets could be differentiated from these connectors. In real life, objects often become partially occluded. This raises the question as to what extent target-distractor merging is affected by occlusion. Experiment 4 addressed this issue. It was found that partially occluding the connectors that joined the targets to the distractors did not improve tracking accuracy compared to a condition where the connectors were not occluded. This indicates that target-distractor merging effects are not affected by occlusion.

**Figure 2:**
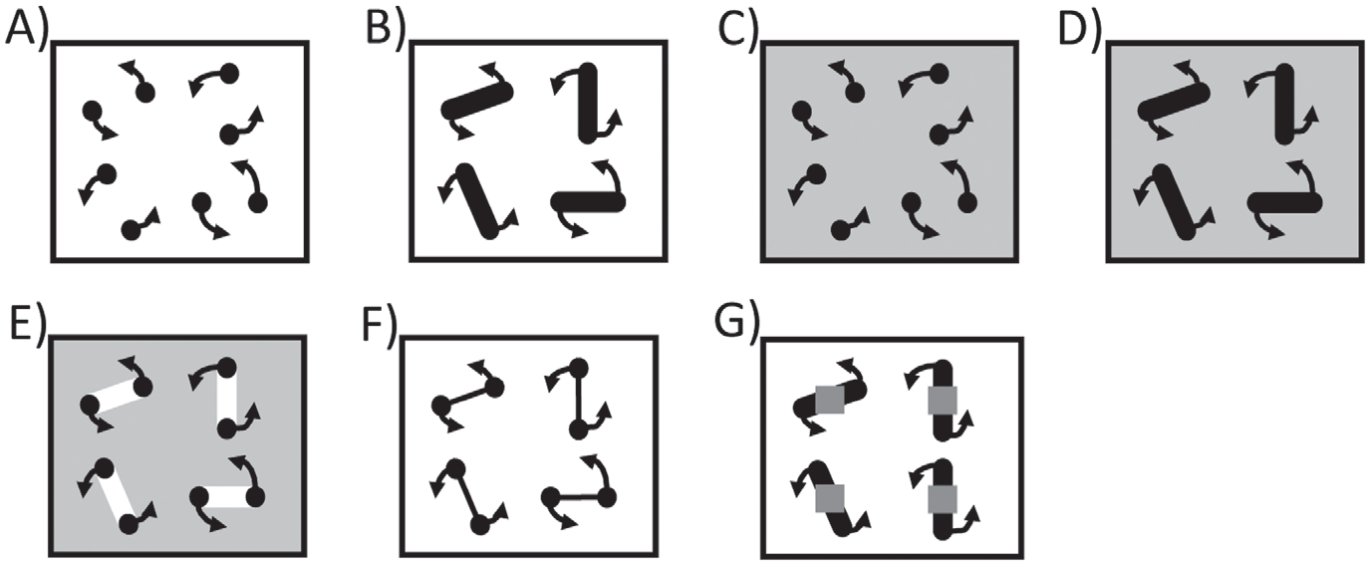
Cartoons of the seven stimuli used in our experiments.

## Experiment 1

The aim of this experiment was to investigate a potential confound in the Scholl et al. experiments [10]. A previous study had shown that object tracking is difficult if the objects continuously expand and contract [12]. We therefore wished to investigate whether the decrement in tracking accuracy in the rubber bands condition (i.e. a target-distractor merged condition) of Scholl et al. was caused by the continuous expanding and contracting that the target-distractor oblongs underwent. To do this, we replicated Scholl et al.’s experiment, but kept the separation between each target-distractor pair constant in both the unconnected baseline condition and in the merged condition. A secondary concern was that Scholl et al. used line drawings. Whilst the target-distractor pairs were in motion, the connectors seemed to be non-rigid, often appearing “gooey” (Scholl et al., p. 172). We therefore used solid shapes in our experiments, so we could more closely imitate objects in real life.

### Method

#### Participants

There were fifteen participants (age range 18–26, 5 male). They all had normal or corrected-to-normal visual acuity and were not colorblind. All observers provided informed written consent and the study was approved by the Department Human Ethics Advisory Group in the Department of Psychology at the University of Melbourne.

#### Apparatus and stimuli

Subjects viewed a 21-inch CRT monitor at a resolution of 1280 by 1024 pixels at a 85 Hz frame rate at a distance of approximately 60 cm. Stimuli were presented in MATLAB (Mathworks, Natick, MA, USA) using Psychophyics toolbox [13], [14]. The stimuli for the two conditions are cartooned in Figures 2a and 2b.

In the baseline condition, there were eight black disks on a white background (Figure 2a). Each disk subtended 1°. Four of the disks were targets and the other four disks were distractors. Each target disk was paired with a distractor disk and both disks revolved around their common center at a rotational frequency of 0.5 Hz. Each pair changed direction randomly, with a probability of 0.5% per presentation frame, i.e. on average they changed direction every 2.35 seconds. The center-to-center spacing of each target-distractor pair was 3.2°. The four center points, one for each target-distractor pair, occurred at the four corners of an imaginary 8.3° square.

The second condition, referred to as the bars condition, was identical to the baseline condition except that each target was joined to its corresponding distractor by a solid black bar (Figure 2b). There were therefore four bars.

#### Procedure

Each trial lasted 8.5 s, during which time the disks moved continuously. For the first 1.5 s, the targets were highlighted in red. For the remaining 7 s, all the disks were identical. When the trial ended, the observer was asked to identify all four targets. Accuracy was defined as the probability of correctly identifying a target disk. The experiment began with 20 practice trials followed by 100 test trials, evenly divided between the two conditions, presented in a random order.

### Results

**Figure 3:**
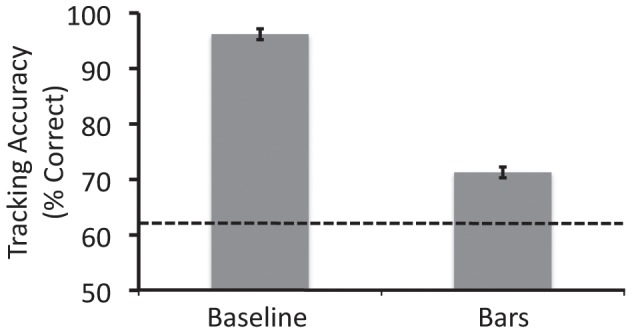
Results from Experiment 1 for the two conditions. The dotted line represents the expected performance level had the observers been able to track only one of the four targets and had to guess where the other three targets were located.

The results are shown in Figure 3. Error bars denote within-observers standard error [15]. Accuracy in the baseline condition was much greater than that in the bars condition, *t*(14) = 12.92, *p*<0.001. This showed that target-distractor merging can substantially reduce tracking accuracy, even when the separation between each target-distractor pair is kept constant.

The dotted line shows the level of performance that would have been expected had the observers only been able to track one target and had to guess the locations of the other three targets. The performance in the bars condition was significantly greater than this chance level of performance, *t*(14) = 22.2, *p*<0.001, indicating that observers were able to track multiple targets in the bars condition, at least in some trials.

## Experiment 2

Experiment 1 demonstrated that target-distractor merging per se can reduce tracking accuracy. However, Scholl et al. demonstrated that this does not always occur [10]. For example, in one of their target-distractor merged conditions, the dumbbells condition, tracking accuracy was barely reduced relative to the unmerged baseline condition. They argued that this occurred because in the dumbbells condition the targets could be differentiated from the connectors, which allowed attention to be confined to the targets.

The motivation of Experiment 2 was to test this hypothesis. Specifically, we investigated whether any manipulation that made it easier to distinguish the targets from the connectors would necessarily reduce the deleterious effects of target-distractor merging and thereby increase tracking accuracy. Scholl et al. had considered only shape manipulations [10]. For example, in their dumbbells condition the targets were distinguished from the connectors by shape differences. At the point where the targets joined the connectors their common boundary had a rapid change in direction thereby demarcating the junction between the target and the connector.

In Experiment 2 we investigated whether other manipulations that caused the targets to be distinct from the connectors would also reduce target-distractor merging effects. Specifically, we tested whether target-distractor merging effects could be eliminated by making the targets a different luminance than the connectors. It has previously been shown that feature differences can have a strong effect on tracking ability. For example, observers find it much easier to confine their attention to a set of moving targets if the targets have a different colour than the distractors [16], [17]. By analogous reasoning we expected observers to find it easier to stop their attention spreading from the targets to the connectors if the targets and connectors had different luminances.

### Method

#### Participants

There were fifteen participants (age range 18–25, 3 male). They all had normal or corrected-to-normal visual acuity and were not colorblind. As before, all observers provided informed written consent and the study was approved by the Department Human Ethics Advisory Group in the Department of Psychology at the University of Melbourne.

#### Apparatus and stimuli

The apparatus was the same as that in Experiment 1. In total there were three conditions. The baseline condition and the bars condition were identical to those of Experiment 1 except that the background was mid-gray instead of white (Figures 2c and 2d). The third condition was the luminance condition, shown in Figure 2e. This condition was identical to the bars condition (Figure 2d) except that the connecting bars were white. Thus, the targets could be differentiated from the connectors by luminance differences, so according to the Scholl et al. hypothesis [10], the observes should have been able to better confine their attention to the targets, with the result that the tracking accuracy in the luminance condition should have been approximately equal to that in the baseline condition.

#### Procedure

As before, each trial lasted 8.5 s, during which time the disks moved continuously. Each pair changed direction randomly, with a probability of 0.5% per presentation frame, i.e. on average they changed direction every 2.35 seconds. For the first 1.5 s, the targets were highlighted in red. For the remaining 7 s, all the disks were identical. When the trial ended, the observer was asked to identify all four targets. Accuracy was defined as the probability of correctly identifying a target disk. The experiment began with 30 practice trials followed by 150 test trials, evenly divided between the three conditions, presented in a random order.

### Results

**Figure 4:**
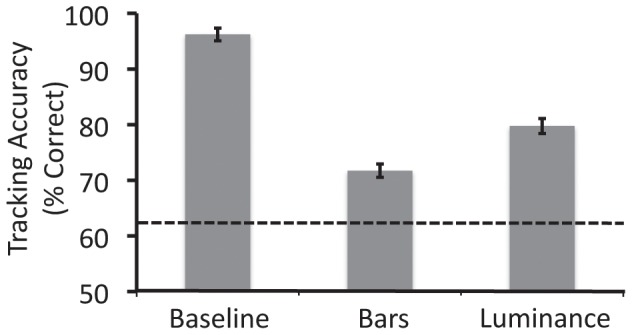
Results from Experiment 2.

The results are shown in Figure 4. Although tracking accuracy in the luminance condition was greater than that in the bars condition, *t*(14) = 14.79, *p*<0.001, it was much less than that in the baseline condition, *t*(14) = 16.95, *p*<0.001. Thus, even though the targets could be differentiated from the connecting bars, a decrement in tracking accuracy was still observed.

## Experiment 3

In the previous experiment we had expected tracking accuracy in the luminance condition to be equal to that in the baseline condition. We were therefore surprised to discover that it was much worse (80% versus 96%). Conversely, in Scholl et al. accuracy in their dumbbells condition was much more similar to their baseline condition (84% versus 92%) [10]. Before we can take this as evidence that luminance differences are much less effective at ameliorating the deleterious effects of target-distractor merging, we need to first verify that we can replicate the results of the Scholl et al. dumbbells condition. This was the motivation for Experiment 3.

### Method

#### Participants

There were fifteen participants (age range 18–25, 6 male). They all had normal or corrected-to-normal visual acuity and were not colorblind. As before, all observers provided informed written consent and the study was approved by the Department Human Ethics Advisory Group in the Department of Psychology at the University of Melbourne.

#### Apparatus and stimuli

The apparatus was the same as that in Experiment 1. In total there were three conditions. The first two conditions were identical to those of Experiment 1 (cf. Figures 2a and 2b). We repeated these conditions so that we could compare performance in these conditions to performance in our third condition using within-subject comparisons as previous research has shown considerable inter-subject variability in tracking performance [18]. The third condition was the dumbbells condition (Figure 2f). This condition was identical to the baseline condition (Figure 2a) except that each target was joined to a distractor by a thin black line (width  = 0.06°) so that the resultant object looked like a dumbbell.

#### Procedure

As before, each trial lasted 8.5 s, during which time the disks moved continuously. Each pair changed direction randomly, with a probability of 0.5% per presentation frame, i.e. on average they changed direction every 2.35 seconds. For the first 1.5 s, the targets were highlighted in red. For the remaining 7 s, all the disks were identical. When the trial ended, the observer was asked to identify all four targets. Accuracy was defined as the probability of correctly identifying a target disk. The experiment began with 30 practice trials followed by 150 test trials, evenly divided between the three conditions, presented in a random order.

### Results

**Figure 5:**
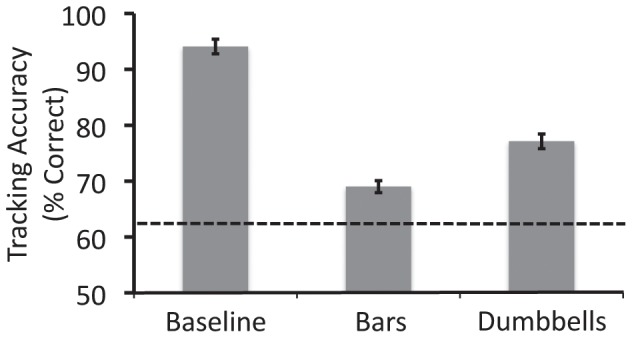
Results from Experiment 3.

The results are shown in Figure 5. Although accuracy in the dumbbells condition was significantly greater than that in the bars condition, *t*(14) = 3.996, *p* = 0.001, it was still much less than that in the baseline condition, *t*(14) = 7.121, *p*<0.001.

## Experiment 4

The previous three experiments have demonstrated that target-distractor merging effects are very robust. They occur even when the separation between the targets and distractors is held constant and continue to occur even when the targets can be differentiated from the connectors that join them to the distractors. As object tracking research moves towards studying more realistic situations where observers need to prioritize tracking particular parts of objects it will be increasing important to understand the basis of target-distractor merging effects. In realistic scenes objects often become partially occlude. Thus, one immediate question is to what extent target-distractor merging is affected by occlusion. Experiment 4 addresses this issue.

### Method

#### Participants

There were fifteen participants (age range 18–55, 7 male). They all had normal or corrected-to-normal visual acuity and were not colorblind. As before, all observers provided informed written consent and the study was approved by the Department Human Ethics Advisory Group in the Department of Psychology at the University of Melbourne.

### Apparatus and stimuli

The apparatus was the same as that in Experiment 1. As before, there were three conditions. The first two conditions were identical to those of Experiment 1 (Figures 2a and 2b). The third condition was the occluded condition (Figure 2g). This condition was identical to the bars condition (Figure 2b) except that a mid-gray square (2.3°×2.3°) was superimposed over the center of each connector. Crucially, each square was stationary and did not revolve with the connector beneath it. At the end of the experiment the observers were debriefed and each reported perceiving the square to be a separate object from the connector below it. All observers perceived the connectors to be partially occluded by the squares above them and for each connector to amodally complete behind its occluding square. The occluded condition therefore allowed us to investigate the extent to which target-distractor merging effects continued to occur when the connectors were partially occluded.

### Procedure

As before, each trial lasted 8.5 s, during which time the disks moved continuously. Each pair changed direction randomly, with a probability of 0.5% per presentation frame, i.e. on average they changed direction every 2.35 seconds. For the first 1.5 s, the targets were highlighted in red. For the remaining 7 s, all the disks were identical. When the trial ended, the observer was asked to identify all four targets. Accuracy was defined as the probability of correctly identifying a target disk. The experiment began with 30 practice trials followed by 150 test trials, evenly divided between the three conditions, presented in a random order.

### Results

**Figure 6:**
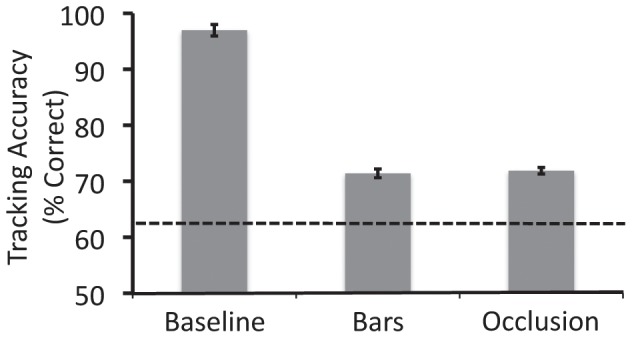
Results from Experiment 4.

The results are shown in Figure 6. Performance in the occluded condition was much worse than performance in the baseline condition, t(14) = 16.9, p<0.001. There was no significant difference between the occluded condition and the bars condition, *t*(14) = 0.511, *p* = 0.62. This shows that target-distractor merging is not significantly affected by occlusion in these particular circumstances.

## Discussion

Scholl et al. have previously shown that connecting targets to distractors can make tracking the targets much more difficult [10]. Each target-distractor pair was perceived to be a single object, making it hard to maintain attention on just the targets. However, in the Scholl et al. study the target-distractor pairs continuously changed length which in itself would have made tracking difficult [12]. Experiment 1 investigated whether the tracking decrement reported by Scholl et al. was due solely to length changes. It was found that even when the length of the target-distractor pairs was kept constant, tracking performance in the target-distractor merged condition was still less than that in the unmerged baseline condition. This showed that target-distractor merging effects per se can reduce tracking accuracy.

Scholl et al. had previously reported that in their target-distractor merged condition, known as the rubber bands condition, observers were only able to track on average one target at a time [10]. However, we found that in our target-distractor merged condition, the bars condition, tracking accuracy was significantly better than this. It is possible that the extra decrement in tracking accuracy observed by Scholl et al. was due to their stimuli continuously changing length [12].

Having confirmed that target-distractor merging per se can reduce tracking accuracy, we then investigated under what circumstances this occurs. Scholl et al. ran eight target-distractor merged conditions [10]. In half of them tracking accuracy was very poor, whereas in the remaining conditions tracking accuracy was high. Scholl et al. hypothesized that target-distractor merging effects do not occur, or at least are greatly reduced, when the targets can be differentiated from the connectors that join them to the distractors. Experiments 2 and 3 tested this hypothesis. In the luminance condition of Experiment 2 the targets were black and the connectors were white so the targets could be differentiated from the connectors. Conversely, in the bars condition, the targets and connectors were the same colour, preventing the targets from being differentiated from the connectors. Although the tracking accuracy in the luminance condition was slightly higher than that in the bars condition, it was still much less than in the baseline condition, whereby the targets were not connected to the distractors. Thus, target-distractor merging effects occurred even when the targets could be readily distinguished from the connectors.

The above result is surprising considering that previous research has shown that featural differences can effectively guide attention. For example, Makovski and Jiang [16], [17] showed that observers find it easier to confine attention to moving targets when the targets have a different colour than the distractors. By analogous reasoning, we expected that making the targets a different luminance to the connectors would make it easier for observers to confine their attention to the targets. Consequently, we had expected tracking accuracy in the luminance condition to be equal to that in the baseline condition where the targets were not physically connectors to the distractors.

It was possible that the above result was unique to luminance and would not hold if we were to make the targets differentiable from the connectors in other ways. Consequently, in Experiment 3 we decided to retest the Scholl et al. differentiability hypothesis by making the targets differentiable from the connectors based on shape differences [10]. Scholl et al. had previously shown that if the connectors joining the targets to the distractors were thinner than the targets and the distractors (i.e. their “dumbbells” condition shown in Figure 1c), observers could readily differentiate the targets from the connectors and tracking accuracy was high. Experiment 3 repeated the Scholl et al. dumbbells experiment to see if we would get a similar result.

Although tracking accuracy in our dumbbells condition was higher than in the bars condition where the targets could not be differentiated from the connectors, it was still much less than in the unmerged baseline condition. In our experiments, it seems that making the targets differentiable from the connectors reduces the decrement in tracking performance associated with target-distractor merging but does not eliminate it.

We hasten to add that we do not claim that target-distractor merging always reduces tracking accuracy. Indeed, in four of the Scholl et al. merged conditions tracking accuracy was either equal to or only slightly less than that in the unmerged baseline condition [10]. It seems that in these conditions the connectors between the targets and distractors appeared insubstantial and “gooey”, causing each target-distractor pair to appear to be two separate objects (Scholl et al., page 172). Conversely, in our experiments the connectors always appeared solid and rigid. This suggests that target-distractor merging effects occur only when each target-distractor pair is *perceived* to form a single object. If this criterion is not met, it seems that tracking accuracy will not be substantially reduced, even if the targets are physically connected to the distractors.

It is unclear why in our experiments observers have difficulty confining their attention to the targets when they are physically connected to the distractors, even when the targets could be differentiated from the connectors that joined them to the distractors. One possibility is that attention has a tendency to spread throughout objects. Thus, attending to one part of an object would automatically cause attention to spread to other parts of the same object.

Egly, Driver and Rafal [8] provided evidence in support of this spreading hypothesis. In their study, observers viewed the outline of two rectangles. One end of one of the rectangles would be briefly cued and then a target would appear and the subject was to respond as quickly as possible. On 75% of the trials the target would be valid in that it would appear at the location of the cue. On the remaining trials, the target would appear elsewhere, so be invalid. On invalid trials, observers were quicker to respond to the target when it appeared within the same rectangle that was cued than when it appeared at an equal distance from the cue but in a different rectangle. This same-object advantage suggests that attention spread throughout the cued rectangle.

Since the publication of this article there has been an ongoing debate as to what conditions are necessary for attention spreading to occur [19], [20]. The most recent evidence suggests that attention will necessarily spread to all areas that are perceived to be part of the same object [19]. If true, this would explain why our observers had difficulty maintaining their attention on the targets when they were perceived to be connected to the distractors, even when the targets could be differentiated from the connectors.

Most theories of object tracking would not seem to be able to predict these results. For example, it has been suggested that objects could be tracked in a serial manner [11], [21]–[23]. According to such theories, each target is attended in turn, one at a time. Every time a target is attended its location is remembered. When it becomes time to reattend a given target, its previously remembered location is used to relocated it, usually by assuming that whichever object is closest to the target’s previously remembered location is in fact the target. These theories would seem to predict that connecting targets to distractors should not decreases tracking ability, providing the targets could still be differentiated from the background and the connectors. Consequently, serial accounts would presumably predict tracking accuracy in our luminance and dumbbells condition to be equal to the tracking accuracy in the baseline condition. Contrary to this, accuracy was much greater in the baseline condition.

To the best of our knowledge there is only one model of object tracking that can currently explain why tracking accuracy drops when targets are physically connected to distractors as occurs in target-distractor merging. Kazanovich and Borisyuk [24] proposed a connectionist model of object tracking that contains multiple layers. Each layer comprises an array of oscillators, referred to as peripheral oscillators. Each peripheral oscillator receives input from a small region of visual space and those oscillators that receive input from the same object synchronize their oscillations. When an object is attended, those peripheral oscillators that receive input from the attended object synchronize their oscillations with a central oscillator. In any one layer, reciprocal inhibitory connections ensure that only one group of peripheral oscillators can be synchronized with a central oscillator at any one time. Thus, each layer can attend, and thus track, at most one object.

Because all oscillators that receive input from the same object synchronize their oscillations, all parts of the object are necessarily attended equally. Thus, even if attention is initially directed to just part of an object, the model predicts that eventually all parts of the object will be attended equally. Consequently, the model is not able to track just part of an object. In this way the model can explain the drop in tracking accuracy that occurs when targets are physically connected to distractors. Unfortunately, the model’s prediction is too extreme. The model predicts that it should never be possible to track just part of an object, whereas in Experiment 1 performance in the target-distractor merged condition, the bars condition, was significantly above chances levels. Furthermore, the model cannot readily explain why certain manipulations can partially ameliorate the effects of target-distractor merging. For example, the model cannot explain why in Experiment 3 tracking accuracy is higher in the dumbbells condition than in the bars condition. According to the model, in neither condition will observers be able to confine their attention to just one end of each object. Thus, although highly promising, this model will need to be developed further before it can provide a full account of our data.

### Conclusions

In this paper we have presented evidence that it is hard to track targets that are physically connected to distractors. We have shown that this occurs when the separation between the targets and distractors is held constant and continue to occur even when the targets can be differentiated from the connectors that join them to the distractors. To the best of our knowledge, currently only one model of object tracking can explain why target-distractor merging reduces tracking accuracy [24], but even this model cannot explain all aspects of our data.
